# Molecular epidemiology of tuberculosis in Tasmania and genomic characterisation of its first known multi-drug resistant case

**DOI:** 10.1371/journal.pone.0192351

**Published:** 2018-02-21

**Authors:** Sanjay S. Gautam, Micheál Mac Aogáin, Louise A. Cooley, Greg Haug, Janet A. Fyfe, Maria Globan, Ronan F. O’Toole

**Affiliations:** 1 School of Medicine, University of Tasmania, Hobart, Tasmania, Australia; 2 Department of Clinical Microbiology, Trinity College Dublin, Dublin, Ireland; 3 Royal Hobart Hospital, Hobart, Tasmania, Australia; 4 Launceston General Hospital, Launceston, Tasmania, Australia; 5 Mycobacterium Reference Laboratory, Victoria Infectious Diseases Reference Laboratory, Peter Doherty Institute, Melbourne, Victoria, Australia; Indian Institute of Technology Delhi, INDIA

## Abstract

**Background:**

The origin and spread of tuberculosis (TB) in Tasmania and the types of strains of *Mycobacterium tuberculosis* complex (MTBC) present in the population are largely unknown.

**Objective:**

The aim of this study was to perform the first genomic analysis of MTBC isolates from Tasmania to better understand the epidemiology of TB in the state.

**Methods:**

Whole-genome sequencing was performed on cultured isolates of MTBC collected from 2014–2016. Single-locus variant analysis was applied to determine the phylogeny of the isolates and the presence of drug-resistance mutations. The genomic data were then cross-referenced against public health surveillance records on each of the cases.

**Results:**

We determined that 83.3% of TB cases in Tasmania from 2014–2016 occurred in non-Australian born individuals. Two possible TB clusters were identified based on single locus variant analysis, one from November-December 2014 (*n* = 2), with the second from May-August 2015 (*n* = 4). We report here the first known isolate of multi-drug resistant (MDR) *M*. *tuberculosis* in Tasmania from 2016 for which we established its drug resistance mutations and potential overseas origin. In addition, we characterised a case of *M*. *bovis* TB in a Tasmanian-born person who presented in 2014, approximately 40 years after the last confirmed case in the state’s bovids.

**Conclusions:**

TB in Tasmania is predominantly of overseas origin with genotypically-unique drug-susceptible isolates of *M*. *tuberculosis*. However, the state also exhibits features of TB that are observed in other jurisdictions, namely, the clustering of cases, and drug resistance. Early detection of TB and contact tracing, particularly of overseas-born cases, coordinated with rapid laboratory drug-susceptibility testing and molecular typing, will be essential for Tasmania to reach the World Health Organisation’s TB eradication goals for low-incidence settings.

## Introduction

There are few descriptions in the literature describing the epidemiology of tuberculosis (TB) in Tasmania or the types of strains of *Mycobacterium tuberculosis* present in its population. Tasmania is a small island state in Australia with approximately 0.5 million people [1]. European settlement of its capital, Hobart, began when it was founded as a penal colony in 1803 by Colonel David Collins who came to Australia with the First Fleet in 1788. The earliest documented evidence of TB in Tasmania comes from Collins who reported that among 36 ill people in his Hobart settlement in 1804, one had consumption [2]. The first recorded deaths from TB in the Tasmanian Aboriginal population occurred between 1835 and 1838 on Flinders Island, situated to the north east of the main Tasmanian island, where they had been relocated from 1830 [2]. The burden of TB in the state increased until reaching a peak of 248 cases in 1940 that corresponded to an incidence rate of 103.8/100,000 in Tasmania compared to the national incidence rate of 59.3/100,000 in Australia at that time [3].

Today, Tasmania is considered a low TB-burden state with an incidence rate of 1.7/100,000 persons compared to 5.7/100,000 nationally in 2014 [4]. There are a number of features of TB in the state that are of interest. Firstly, TB in Tasmania has been considered to consist of isolated unique cases that have been imported from other jurisdictions. Secondly, the state has been free of multi-drug resistant forms of TB. Thirdly, as part of the Brucellosis and Tuberculosis Eradication Campaign (BTEC) in Australia, bovine TB disease was eradicated from Tasmanian cattle herds in 1975 thus, eliminating the primary source of human cases of *M*. *bovis* TB in the state [5].

In this study, we performed an in-depth analysis of the types of MTBC strains isolated in Tasmania between 2014 and 2016 using whole-genome sequencing. We then correlated genomic information with public health surveillance data to better define the epidemiology of TB in Tasmania.

## Methods

### Study design

Samples from 18 cultured isolates collected in Tasmania from 2014 to 2016, inclusive, were available for this study. This represents 62.1% of total TB notifications that occurred during this time period (n = 29) [6]. Diagnostic laboratory and clinical data were obtained from the Royal Hobart Hospital, Launceston General Hospital, and the Victorian Infectious Diseases Reference Laboratory (VIDRL). Samples were sent to the School of Medicine, University of Tasmania, for next generation sequencing and whole-genome bioinformatics analysis. Ethics approval for this study was obtained from the Tasmanian Health and Medical Human Research Ethics Committee (H0016214). A waiver of consent was acquired as the study was an observational non-interventional analysis of MTBC isolates and de-identified data that were obtained from routine laboratory testing.

### Sample processing, culture, and drug susceptibility testing

Specimens from patients suspected of having tuberculosis were cultured using both solid (Brown and Buckle agar, Löwenstein-Jensen agar) and liquid media (Mycobacterial Growth Indicator Tubes (MGIT)) in accordance with standard protocols for mycobacterial growth [7]. Ziehl-Neelsen staining, TB MPT64 antigen test (Standard Diagnostics Bioline TB MPT64 antigen test) and TB PCR (GenXpert, Cepheid) were performed on positive cultures. The isolates were supplied to a reference laboratory for further characterisation. Drug-susceptibility testing was performed using the MGIT system [7].

### Genomic DNA isolation

1.5 mL of heat-inactivated mycobacterial cultures were centrifuged at 8,000 rpm for 3 minutes at room temperature. The cell pellet was resuspended in 200 μL phosphate-buffered saline and treated with 25 μL of 10 mg/mL lysozyme and incubated at 37°C for 1 hour followed by 95°C for 15 minutes. 30 μL proteinase K (10 mg/mL) were added and the sample was incubated at 55°C for 30 minutes. A Qiagen DNeasy Blood and Tissue kit was then used to extract mycobacterial genomic DNA as per the manufacturer’s instructions and the DNA was eluted with 200 μL of Buffer AE. 1 μL of RNase A (7000 units/mL, Qiagen) was added to 50 μL of genomic DNA eluent and incubated at room temperature for 1 hour. The genomic DNA was further purified using a High Pure PCR Template Preparation Kit as per the manufacturer’s instructions (Roche) and quantified using the Quant-iT Qubit^™^ dsDNA HS Assay Kit (Thermo Fisher Scientific).

### Whole genome sequencing and data analysis

Purified genomic DNA was tagged and amplified using a Nextera^®^ XT DNA Library Preparation Kit and Nextera^®^ XT Index Kit as per the manufacturer’s (Illumina) instructions. The libraries generated were cleaned using Agencourt AMPure XP beads, normalized and then pooled. The concentration of the pooled library was determined by qPCR using a KAPA Library Quantification Kit. 15 pM of the pooled library were loaded into a MiSeq Reagent Kit v2 cartridge and run on an Illumina MiSeq instrument generating paired-end reads of 150 base pairs (bp) (maximum). The fastq sequence files were collected and analysed using Geneious software suite (R 9.5) [8]. Paired-end reads were trimmed (error probability limit of 0.05) and then mapped (random multiple base matches) to the publicly-available annotated genome of *M*. *tuberculosis* reference strain H37Rv (accession number NC_000962.3) [9] using a maximum variant p-value of 10^−6^ when exceeding 65% bias. Single-locus variations (SLV) were called at a minimum variant frequency of 95% and a minimum mean genome coverage of 20, and were annotated as previously described [10]. Mycobacterial lineage was predicted with TB Profiler [11] and mutations associated with drug resistance were detected using the PhyResSE database [12] followed by manual checking of the sequence. A maximum likelihood phylogenetic inference tree was built in PhyML using the generalised time reversible (GTR) substitution model [13].

### TB epidemiological analysis

A threshold of ≤5 single nucleotide polymorphisms (SNPs) between *M*. *tuberculosis* isolates has previously been proposed as an indicator of recent TB transmission between patients, while >12 SNP differences between isolates has been considered as evidence against recent transmission [14–16]. SNP distances between TB isolates can be affected by factors such as time between patient sampling, local TB incidence, and homogeneity of *M*. *tuberculosis* strains in some regions [17, 18]. Therefore, for isolates that were within 5 SNP differences of one another, additional epidemiological data were used. The definition of a possible cluster was based on the National Tuberculosis Advisory Committee of Australia’s guidelines which state that “A ‘possible cluster’ will be any 2 or more active cases with the same genotype as defined by the method used where temporal and geospatial association is plausible but no direct epidemiological link is identified” [19]. Each *M*. *tuberculosis* isolate in this work was characterised based on SNP differences to other members of the same global lineage, and the presence of spatiotemporal links between cases. Furthermore, *in silico* spoligotyping of the isolates was performed using the Total Genotyping Solution for TB (TGS-TB) database [20] and compared with published data on MTBC genotypes in the patient’s country/region of origin.

## Results

### Relative distribution of MTBC lineages in Tasmania

Cases were 72.2% male (*n* = 13), 27.8% female (*n* = 5) (Table 1). The age of patients at date of specimen collection ranged from 3 months to 70 years of age with a mean age of 33.6 years. 77.7% (*n* = 14) of cases were pulmonary and 22.2 (*n* = 4) were extra-pulmonary. 83.3% of cases (*n* = 15) were non-Australian born and 16.7% (*n* = 3) were Australian-born (Table 1).

**Table 1:** Demographic and specimen information for tuberculosis cases (*n* = 18) in Tasmania from 2014 to 2016. Demographic variables on the TB cases and specimen types were recorded. Cases were 72.2% male and 27.8% female. The mean TB patient age was 33.6 years (range 0–70 years).

Isolate Name	Age Range of Patient (years)	Specimen Type	Year of Specimen Collection	MTBC Lineage	Patient Country of Origin
RHH2	20–39	Sputum	2015	1	Philippines
RHH3	≥60	Sputum	2015	3	Nepal
RHH4	20–39	Sputum	2016	4	Thailand
RHH5	20–39	Paraspinal aspirate	2015	1	Myanmar/Malaysia
RHH6	20–39	Osteomyelitis	2014	3	Nepal
RHH7	40–59	Sputum	2014	4	New Zealand
RHH8	20–39	Sputum	2016	2	Malaysia
RHH9	<5	Gastric aspirate	2014	4	New Zealand
RHH10	20–39	Sputum	2016	3	Nepal
RHH11	40–59	Sputum	2015	3	Nepal
RHH12	<5	Gastric aspirate	2016	1	Philippines
RHH13	20–39	Sputum	2015	3	Nepal
RHH14	<5	Gastric aspirate	2015	3	Nepal
RHH15	≥60	Sputum	2014	4	Australia
TTB1	≥60	Urine	2016	*M*. *bovis* BCG	Australia
TASMDR1	20–39	Tissue	2016	2	Vietnam
TTB3	20–39	Sputum	2016	1	Philippines
TASMB14	≥60	Sputum	2014	*M*. *bovis*	Australia

MTBC, *Mycobacterium tuberculosis* complex

**Fig 1:**
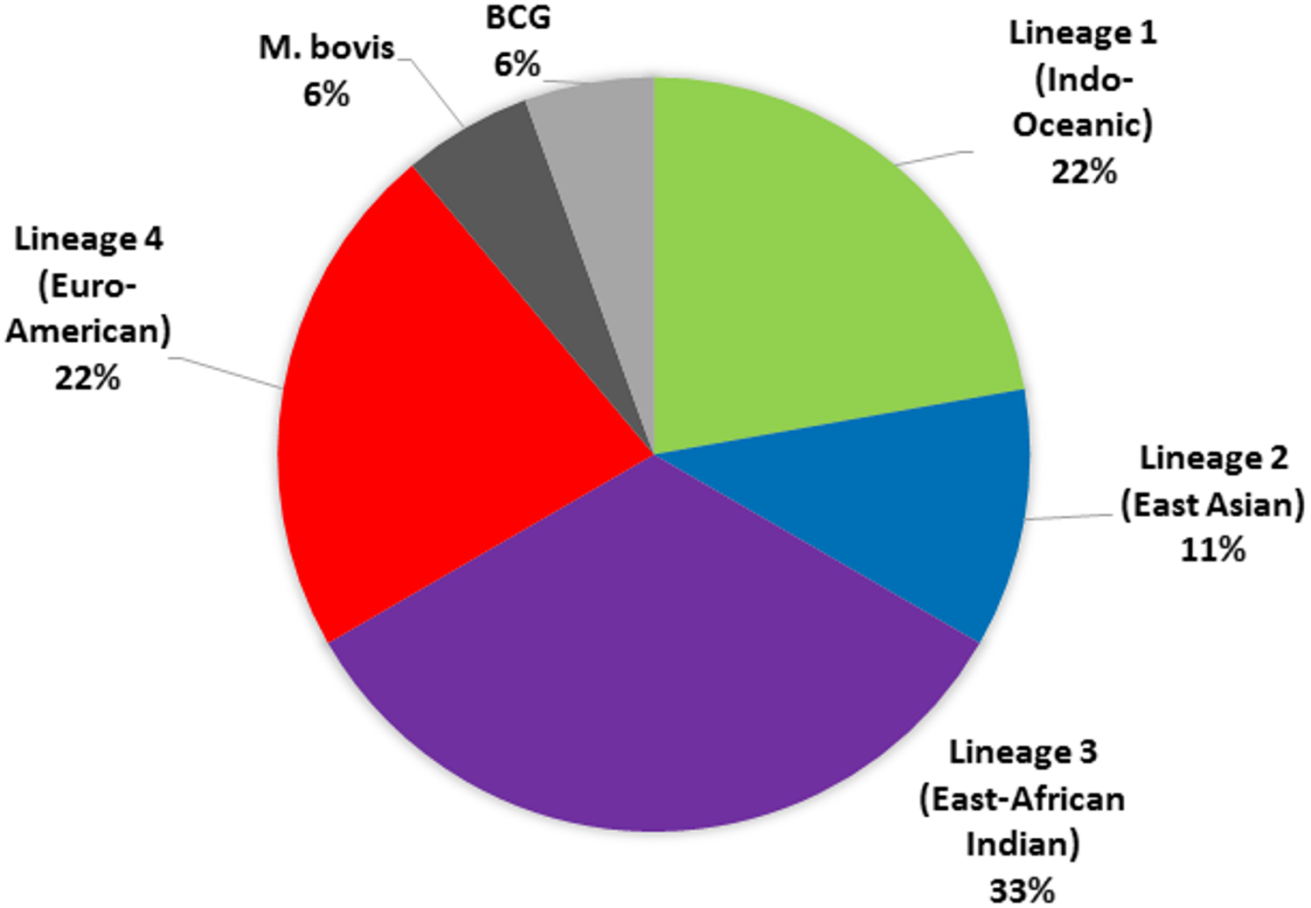
Relative frequency of *Mycobacterium tuberculosis* complex (*n* = 18) isolates in Tasmania from 2014 to 2016.

Whole-genome sequence data were obtained for the 18 TB isolates analysed from 2014–2016. The phylogenetic lineage of each isolate was determined using the PhyResSE and TB Profiler databases [11, 12]. The most common lineage among the samples analysed was the East-African Indian Lineage 3 (*n* = 6, 33.3%) followed by the Euro-American Lineage 4 (*n* = 4, 22.2%), Indo-Oceanic Lineage 1 (*n* = 4, 22.2%), and East-Asian Lineage 2 (*n* = 2, 11.1%) (Fig 1). In addition, cases of TB due to *M*. *bovis* (*n* = 1) and *M*. *bovis* BCG (*n* = 1) were recorded in 2014 and 2016, respectively.

### Molecular epidemiological clustering of TB cases

**Fig 2:**
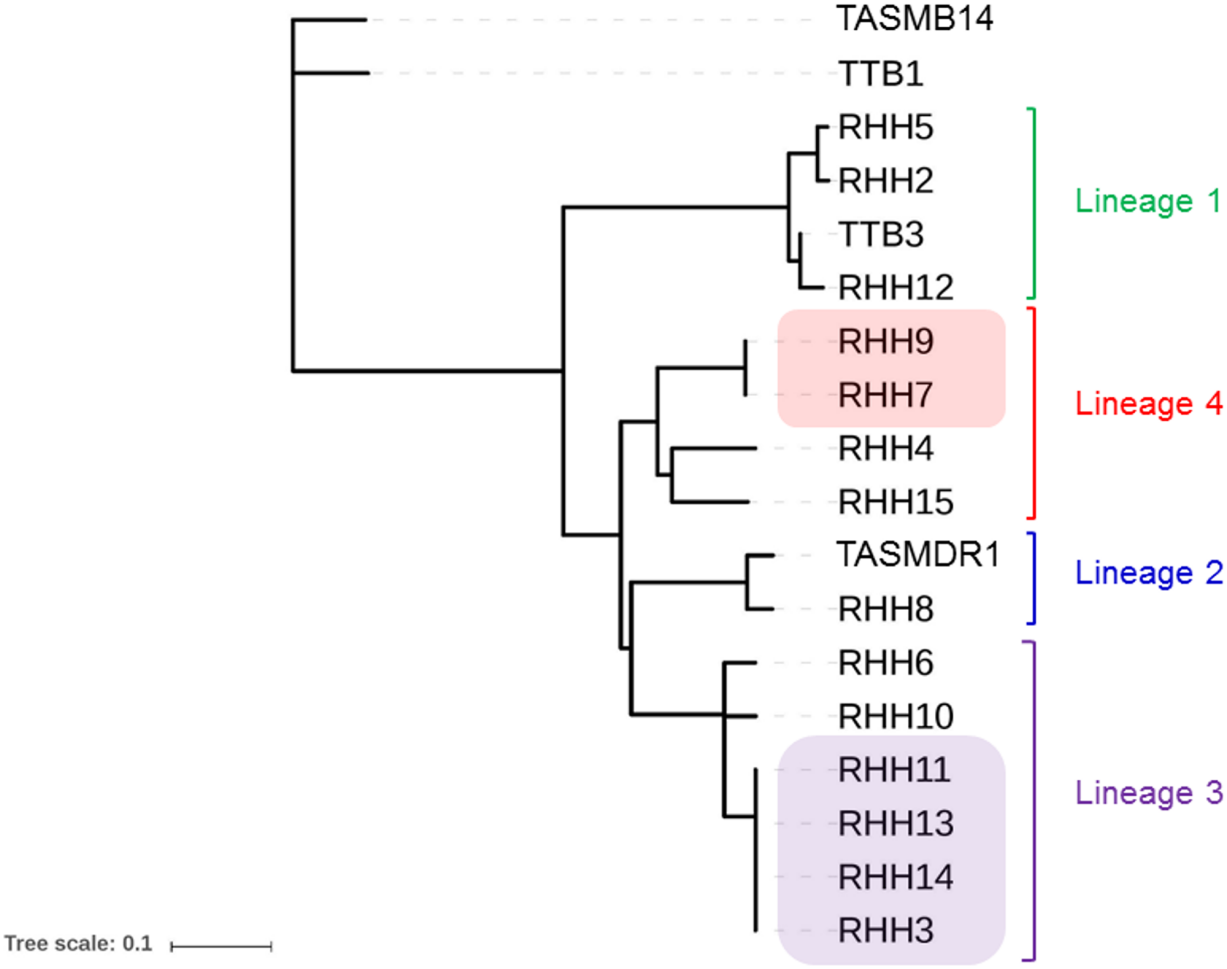
Phylogenetic relationship of *Mycobacterium tuberculosis* complex isolates in Tasmania from 2014 to 2016. TASMB14 and TTB1 constitute isolates of *M*. *bovis* and *M*. *bovis* BCG, respectively. The Gagneux lineage numbers are indicated for the other isolates. Lineage 2 isolate TASMDR1 is a multi-drug resistant isolate of *Mycobacterium tuberculosis*. Lineage 3 isolates RHH3, 11, 13 and 14 are identical (zero SNP differences with respect to one another) and form an epidemiological cluster as do Lineage 4 isolates RHH7 and RHH9. The phylogenetic tree was built using PhyML (Generalised Time Reversible substitution model).

A maximum likelihood phylogenetic inference tree generated using PhyML revealed grouping of the different isolates into specific clades which were in agreement with the lineage analysis performed using the PhyResSE and TB Profiler databases (Fig 2). In addition, the phylogenetic tree revealed possible genetic clusters of isolates within Lineages 3 and 4.

**Fig 3:**
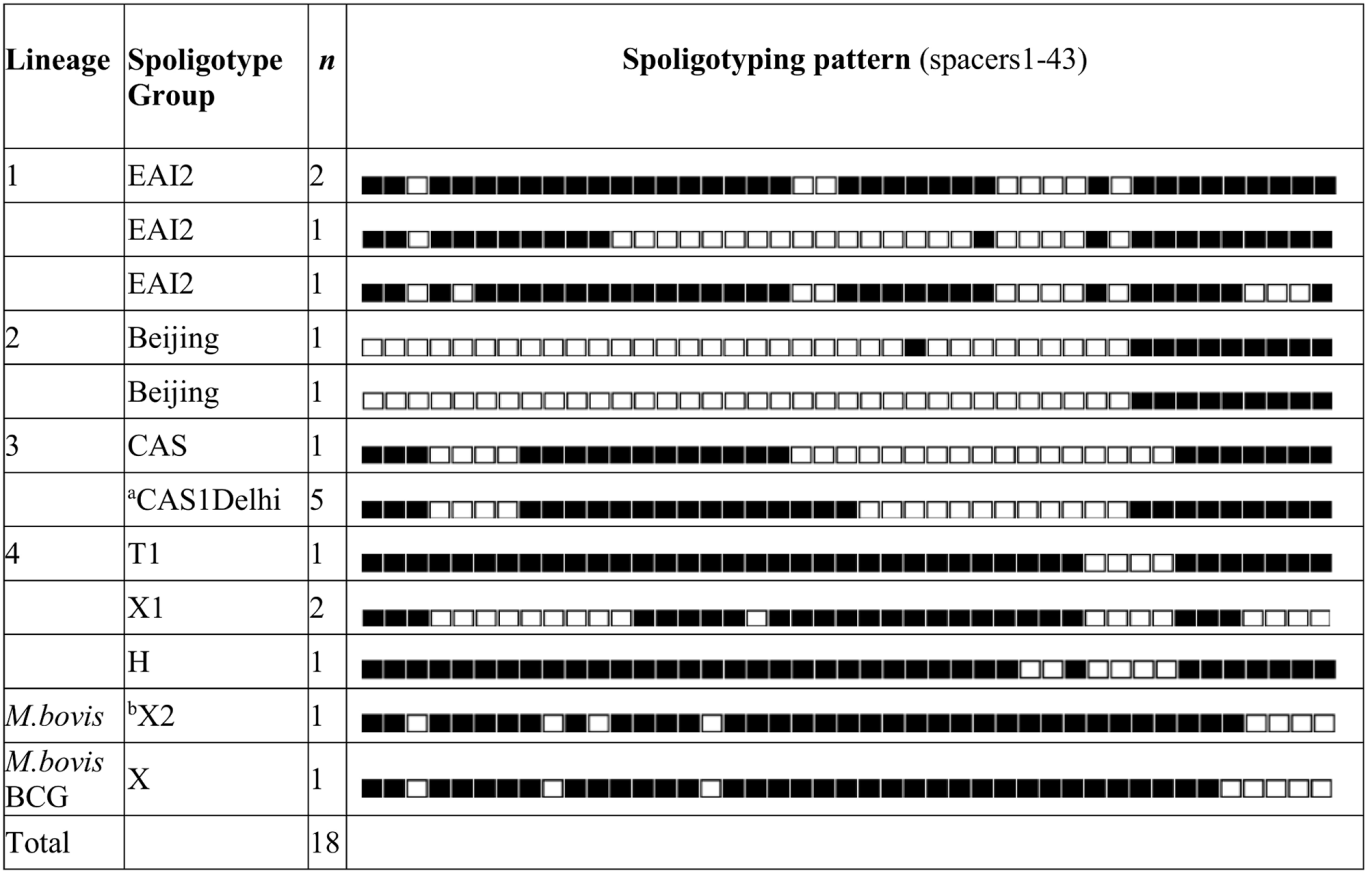
Distribution of *in silico* generated spoligotypes across the culture-positive Tasmanian TB isolates analysed from 2014–2016. ^a^The *in silico* derived spoligotype of the four Tasmanian Lineage 3 cluster isolates (RHH3, RHH11, RHH13, RHH14) and a fifth Lineage 3 isolate (RHH10) matched Spoligotype International Type 26 of the CAS1_Delhi spoligotyping family which accounted for approximately 50% of Lineage 3 *M*. *tuberculosis* isolates in Nepal in a previous analysis [21]. ^b^The *in silico* derived spoligotype of the *M*. *bovis* isolate (TASMB14) matches that of human *M*. *bovis* cases that were reported in other Australian states/territories between 1977 and 1989 [22].

The Lineage 3 cluster consisted of four isolates (RHH3, RHH11, RHH13, and RHH14) which exhibited zero SLV differences with respect to one another. Based on the proposed threshold of ≤5 SNPs, from Walker and others [14, 15], this is indicative of recent transmission. The isolates were collected in Tasmania from May to August 2015 from drug-susceptible cases of pulmonary TB. The patients were household contacts and were originally from Nepal. A previous analysis of 261 *M*. *tuberculosis* isolates collected in Nepal from pulmonary TB patients between August 2009 and August 2010 using spoligotyping and real-time PCR analysis of SNPs found that the most frequent *M*. *tuberculosis* lineage was Lineage 3 (40.6%) [21]. The *in silico* spoligotype of all four Tasmanian Lineage 3 cluster isolates matched Spoligotype International Type 26 of the CAS1_Delhi spoligotyping family which was found to account for approximately 50% of Lineage 3 *M*. *tuberculosis* isolates in the previous analysis of Nepal TB cases (Fig 3) [21].

The Lineage 4 cluster comprised two isolates (RHH7 and RHH9) which exhibited zero SLV differences with respect to one another. The isolates were collected in Tasmania from November to December 2014 from drug-susceptible cases of pulmonary TB. The patients were household contacts and were originally from New Zealand. From an earlier study involving 487 MTBC isolates collected in New Zealand from January 2010 to December 2011, the most prevalent lineage was Lineage 4 which made up 37.8% of TB cases in the general population and 70.5% of cases in the New Zealand-born population [23].

The remaining MTBC isolates (*n* = 12) were phylogenetically unique with respect to one another with >50 SNP differences between them. The closest isolates from this group were isolates TTB3 and RHH12 which differ by 74 single-locus variations and belong to Indo-Oceanic Lineage 1. We do not have epidemiological evidence that these patients formed a probable cluster. Both patients were originally from the Philippines where Lineage 1 is highly dominant among TB isolates [24, 25].

### First confirmed case of MDR-TB in Tasmania

An overseas-born individual tested positive for TB infection in an Interferon Gamma Release Assay (IGRA) test in 2016 but did not exhibit symptoms of TB, had a normal chest x-ray, and was sputum culture negative. The patient presented with developed abdominal pain consistent with colitis later in 2016 and a colon biopsy sample was subsequently taken. The colon tissue specimen was smear-negative but culture-positive for *Mycobacterium tuberculosis*. MGIT based drug-susceptibility testing performed on this extra-pulmonary isolate at VIDRL revealed that it was resistant to isoniazid, rifampicin, ethambutol, pyrazinamide making it the first confirmed case of MDR-TB to have occurred in Tasmania. The isolate was recorded as sensitive to ethionamide, amikacin, capreomycin, kanamycin, ofloxacin and moxifloxacin.

Genomic DNA of the Tasmanian MDR-TB isolate (TASMDR1) was sequenced on an Illumina MiSeq. Paired-end reads were mapped to the *M*. *tuberculosis* H37Rv reference genome by Burrows-Wheeler Alignment producing a mapped-read depth of 73.7-fold, covering 97.36% of the H37Rv genome. A consensus sequence was called using SAMtools generating a 4,320,496-bp draft assembly. With respect to reference H37Rv genome, 1,566 SLVs were detected in the TASMDR1 assembled genome, of which 874 were non-synonymous. An analysis was then performed to identify SLVs which correlated with phenotypic drug resistance. The genome of TASMDR1 displayed single-nucleotide polymorphisms in genes correlating with antimicrobial drug resistance when analysed using the PhyResSE database [12]. These included high confidence mutations in the genes *katG* (aGc/aCc, S315T) and *rpoB* (gAc/gGc, D435G; tCg/tTg, S450L) which are associated with *M*. *tuberculosis* resistance to isoniazid and rifampicin, respectively [26–28] (Table 2).

**Table 2:** Mutations detected in the genome of the TASMDR1 isolate that confer resistance to anti-tubercular drugs. Six mutations that have been associated with anti-tubercular drug resistance were identified. The mutations listed in the *rpoB*, *katG*, *pncA*, and *embB* genes were classified as high confidence SNPs with respect to resistance to rifampicin, isoniazid, pyrazinamide and ethambutol, respectively, by the PhyResSE database [12]. In addition, an A/C substitution was detected at position 514 of the 16S rRNA gene, *rrs* (MTB000019) that is associated with streptomycin resistance [33, 34].

Drug	Gene	Locus Tag	Mutation	Genome Location	Substitution
Rifampicin	*rpoB*	Rv0667	gAc / gGc, tCg /tTg	761110, 761155	D435G, S450L
Isoniazid	*katG*	Rv1908c	aGc / aCc	2155168	S315T
Pyrazinamide	*pncA*	Rv2043c	cCg / cTg	2289057	P62L
Ethambutol	*embB*	Rv3795	Atg / Gtg	4247429	M306V
Streptomycin	*rrs*	MTB000019	A / C	1472359	a514c

Further mutations were detected in the *embB* (Atg/Gtg, M306V) and *pncA* (cCg/cTg, P62L) genes that underlie resistance to ethambutol and pyrazinamide, respectively [29–32] (Table 2). In addition, an additional A/C substitution was detected at position 514 of the 16S rRNA gene, *rrs* (MTB000019) that is associated with streptomycin resistance [33, 34] (Table 2). The TASMDR1 isolate was predicted to belong to East Asian Lineage 2, sub-lineage Beijing, by the PhyResSE and TB Profiler databases [11, 12]. Furthermore, the isolate exhibited a polymorphism in the *mutT2* gene (Gga/Cga, G58R) which is associated with so-called ‘Modern’ Beijing strains [35, 36]. The patient was originally from Viet Nam and had known household contact with an active case of TB that was confirmed in Viet Nam in 2012. The isolate from this 2012 case was recorded as resistant to isoniazid, rifampicin, ethambutol, pyrazinamide and streptomycin from MGIT based drug-susceptibility testing.

### Case of *M*. *bovis* TB in Tasmania

Isolate TASMB14 was collected in Tasmania in 2014 from a sputum specimen taken from a drug-susceptible case of pulmonary TB in an Australian-born person. Risk factors associated with this case included age (≥70 years) and chronic obstructive pulmonary disease co-morbidity. The genome sequence of the isolate revealed that it contains the Rv2043c (*pncA*) polymorphism, Cac/Gac, H57D, and the RD1 region genes Rv3871 to Rv3879c, confirming it as *M*. *bovis*. The *in silico* derived spoligotype of TASMB14 matches that of other human *M*. *bovis* cases that were reported elsewhere in Australia between 1977 and 1989 [22] (Fig 3).

## Discussion

In this study, we provide the first in-depth analysis of the molecular epidemiology of tuberculosis in Tasmania. MTBC isolates collected from culture-positive cases of TB in Tasmania from 2014 to 2016, were examined. The most common lineage detected among the Tasmanian samples analysed was the East-African Indian Lineage 3 (33.3%) followed by the Euro-American Lineage 4 (22.2%), Indo-Oceanic Lineage 1 (22.2%), and the East-Asian Lineage 2 (11.1%) (Fig 1). Individual cases of TB due to *M*. *bovis* and *M*. *bovis* BCG were notified in 2014 and 2016, respectively.

Our whole-genome sequence analyses identified two possible clusters of *M*. *tuberculosis* among the Tasmanian cases, one belonging to Lineage 3 and the other belonging to Lineage 4. The Lineage 3 cluster, consisted of four isolates separated by zero SLVs which is indicative of recent transmission between the patients based on previously-established SNP thresholds [14, 15]. The isolates were collected within a three-month period from household contacts who originated from Nepal. The *in silico* generated spoligotype of the four Lineage 3 cluster isolates matches Spoligotype International Type 26 of the CAS1_Delhi spoligotyping family. This particular spoligotype was common among TB cases in Nepal, constituting approximately 50% of Lineage 3 isolates, and 20% of total TB isolates, in an earlier study (Fig 3) [21].

In this work, we describe the first documented case of MDR-TB in Tasmania. This case was detected in the second half of 2016 in an overseas-born individual who had earlier moved from Viet Nam to Tasmania. The isolate, TASMDR1, which belongs to the East-Asian Lineage 2, was confirmed as being resistant to isoniazid, rifampicin, ethambutol and pyrazinamide in phenotypic drug-susceptibility testing. Furthermore, genome sequencing identified an a514c mutation in the *rrs* locus (MTB000019) that is associated with streptomycin resistance. A household contact of the patient had been diagnosed with pulmonary MDR-TB in Viet Nam in 2012. The isolate from this 2012 case was recorded as resistant to isoniazid, rifampicin, ethambutol, pyrazinamide, and streptomycin in MGIT culture-based drug-susceptibility testing. Based on the equivalent drug-resistance profiles of the two MDR-TB cases, it is likely that the Tasmanian case contracted the MDR strain of *M*. *tuberculosis* from the household contact some time previously and that the infection remained latent until reactivating as extrapulmonary MDR-TB in 2016. A recent study by Fox and colleagues conducted in Viet Nam found that household contacts of patients with MDR-TB have a higher risk of becoming tuberculin-skin test positive and of developing active TB compared to contacts of drug-susceptible TB [37].

While the proportion of TB cases in Australia that are MDR is currently under 2% (22 MDR-TB cases out of 1,263 TB notifications in 2013), the estimated costs associated with treating a case of TB increase substantially when going from drug-susceptible TB (USD$17,000 in the USA, €10,282 in 15 EU countries, per case) to multi-drug resistant TB (USD$134,000 in the USA, €57,213 in 15 EU countries, per case) [38, 39]. It was previously estimated that management of one case of extensively drug-resistant (XDR) TB in 2012 cost Queensland Health in the region of AUD $500,000 [40]. Hence, vigilance will need to be maintained with respect to the tracing of contacts of previous TB cases, especially MDR-TB cases, and the early detection of drug resistance in Tasmanian isolates.

Human TB caused by *M*. *bovis* was reported in Tasmania in 2014, nearly 40 years after the last confirmed case of bovine TB in the state in 1975 [5]. The pulmonary form of disease was diagnosed in a male aged ≥70 years. The source of this infection is unknown but a possibility is reactivation of a latent *M*. *bovis* infection acquired during earlier rural exposure to *M*. *bovis* prior to the elimination of bovine TB disease in Tasmania. The *in silico* derived spoligotype of the isolate, TASMB14, matches that of previously-described human *M*. *bovis* cases that were reported in other Australian states and territories between 1977 and 1989 [22] (Fig 3). As noted in 1999 by Cousins et al., “because of the usual long incubation periods that can occur between infection and development of disease, and because of the possibility of disease reactivation, especially in elderly or immunocompromised patients, human tuberculosis caused by *M*. *bovis* is likely to continue to be diagnosed for many years to come” [41].

In the majority of the Tasmanian cases analysed from 2014 to 2016, 83.3% of patients (*n* = 15) were born overseas. This corresponds with 89.2% and 87.6% of TB notifications nationally recorded in the overseas-born population in 2012 and 2013, respectively [42]. A number of European studies have found that immigrants are not a major source of TB infection for the native-born population [43, 44]. Sandgren and colleagues in their systematic review concluded, that “TB in a foreign-born population does not have a significant influence on TB in the native population in EU/EEA” [45]. In our study, we did not find evidence of transmission of TB from the overseas-born cases to the Australian-born population. Nevertheless, targets have been set for low incidence jurisdictions by the World Health Organisation for the pre-elimination of TB by 2035 (defined as <10 TB cases per million population), and the elimination of TB by 2050 (<1 TB case per million population) [46]. The incidence rate of TB in Tasmania currently sits at approximately 16 per million population (1.6/100,000) [42]. Hence, a 60% drop in TB cases by 2035, and a 95% drop in TB cases by 2050 are required in Tasmania for the state to meet international targets.

A common trend seen in low TB burden countries is decreasing TB in the native-born population and increasing TB in the migrant population as a proportion of total cases [47]. Therefore, bringing TB rates into line with the World Health Organisation’s goals will require efforts to reduce TB incidence in the foreign-born population. A major emphasis in Australia is placed upon pre-immigration screening. Visa applicants who are 11 years or older must undergo a chest x-ray and potentially, other diagnostic tests. If active TB is found, Australian law does not permit the granting of a visa until the applicant has completed treatment and has been declared free of active TB [48]. Pre-entry screening of foreign-born individuals is not specifically designed for the detection of latent tuberculosis infection (LTBI). However, most TB cases among the foreign-born population in industrialised countries are believed to be due to reactivation of LTBI rather than continuation of an existing case of active TB [49, 50]. In addition, molecular epidemiological studies have found a strong association between the lineage of the MTBC strain isolated from a migrant patient and the predominant lineage found in their region of origin [23–25]. In our study, all Lineage 3 (Central Asian (CAS)/Delhi) cases were in individuals from the Indian sub-continent where this lineage is prevalent [51]. It is probable that a number of the overseas-born patients who presented with TB in Tasmania had acquired *M*. *tuberculosis* infection prior to their arrival in the state or in Australia. Therefore, further consideration will need to be given to the management of TB in the migrant population in Tasmania in order to reduce the incidence of the disease in the state.

## Conclusions

In summary, our work provides the first extensive analysis of the molecular epidemiology of tuberculosis in Tasmania. It identified the presence of two phylogenetic clusters of identical isolates of *M*. *tuberculosis* which is indicative of recent transmission of TB among household contacts. In addition, it established the genetic basis of the resistance exhibited by Tasmania’s first confirmed case of MDR-TB. Our study highlights that while the incidence of TB in Tasmania is comparatively low, challenges remain with regard to the management of the disease in the migrant population, particularly from high TB prevalence countries, which will need to be overcome for the state to meet the World Health Organisation’s 2035 and 2050 TB eradication goals.
